# Efficient Delivery of Macromolecules into Human Cells by Improving the Endosomal Escape Activity of Cell-Penetrating Peptides: Lessons Learned from dfTAT and its Analogs

**DOI:** 10.3390/biom8030050

**Published:** 2018-07-11

**Authors:** Jason K. Allen, Dakota J. Brock, Helena M. Kondow-McConaghy, Jean-Philippe Pellois

**Affiliations:** 1 Department of Biochemistry and Biophysics, Texas A&M University, College Station, TX 77843, USA; jkallen@tamu.edu (J.K.A.); dbrock@tamu.edu (D.J.B.); hkondow14@tamu.edu (H.M.K.-M.); 2 Department of Chemistry, Texas A&M University, College Station, TX 77845, USA

**Keywords:** peptide, cell-penetrating peptide, cellular delivery, endosomal escape, membrane leakage, TAT peptide, multimerization, chirality, charge density

## Abstract

Cell-penetrating peptides (CPPs) are typically prone to endocytic uptake into human cells. However, they are often inefficient at escaping from endosomes, which limits their ability to deliver cargos into cells. This review highlights the efforts that our laboratory has devoted toward developing CPPs that can mediate the leakage of endosomal membranes, and consequently gain better access to the intracellular milieu. In particular, we have identified a CPP named dimeric fluorescent TAT (dfTAT) with high endosomolytic activity. We describe how we have used this reagent and its analogs to develop efficient cytosolic delivery protocols and learn about molecular and cellular parameters that control the cell permeation process. Specifically, we discuss how late endosomes represent exploitable gateways for intracellular entry. We also describe how certain features in CPPs, including guanidinium content, charge density, multimerization, chirality, and susceptibility to degradation modulate the activity that these peptidic agents take toward endosomal membranes and cytosolic egress.

## 1. Introduction

Cell-penetrating peptides (CPPs) are often used as delivery agents for cell-impermeable macromolecular cargos of biological or synthetic origin [1,2]. Many CPPs utilize the endocytic pathway to enter cells [3,4]. As a first step, the CPP–cargo conjugates tend to localize inside endosomes following endocytic uptake [5]. At this point, many molecules remain entrapped in the lumen of those organelles, and thereby cannot access the cytosolic space of the cell. Yet, access to the cytosolic space is often a prerequisite for the macromolecular cargo to exert its biological function [6]. Thus, once in the lumen of endosomes, the CPP–cargo conjugates must escape from endosomes into the cytosolic space for successful delivery. Evidence suggests that several CPPs have the ability to mediate endosomal escape. However, it appears that this activity is weak in many cases [7]. Concomitantly, how endosomal escape is mediated is often unclear, thereby limiting our ability to manipulate parameters that may control this process. Together, these problems lead to delivery outcomes that are poor, of variable reproducibility, and unpredictable. Consequently, it is important to develop reagents that mediate endosomal escape and identify key chemical or cellular parameters that can be exploited for improved cytosolic penetration.

A focus of our laboratory has been to develop endosomolytic reagents and use them as models that can reveal how endosomal escape is mediated in living cells. Based on several reports in the literature, we first investigated the membrane-disrupting capabilities of virus-derived peptides [8]. These peptides are amphiphilic and pH-responsive. Therefore, as the lumen of the maturing endosome acidifies, these peptides bind and disrupt membranes [9]. We found that such reagents were relatively efficient at causing endosomal permeation [10]. Yet, their hydrophobicity also makes them prone to aggregation with themselves and with cargos, and therefore difficult to utilize as delivery agents [7,11]. Another approach that we investigated was that of photochemical internalization, which is a strategy that relies on the photooxidation of endosomal membranes [12,13,14]. In particular, similar to other groups, we used fluorescently-labeled CPPs to induce endosomal escape by irradiation with visible light [15,16]. Although very rapid and efficient, we found that this light-induced endosomal escape is accompanied by a rapid burst of calcium release into the cell. This, in turn, leads to mitochondrial permeabilization and cell death, which is an obvious problem [16]. To circumvent the cytotoxic response triggered by the previous constructs, we focused our next design strategy on creating multivalent constructs, which is an idea that had proven successful in the literature for a number of applications [17,18,19,20]. This design involves incorporating multiple copies of a given CPP sequence on a scaffold [21]. Among the various reagents that were developed, the structurally simple CPP dfTAT, or dimeric fluorescent TAT, was identified as an extremely efficient yet non-toxic endosomolytic agent [22]. 

In this review, we describe what we have learned about how dfTAT works and what it does on both a cellular and molecular level. Some lessons may be specific to our reagents, but others may be of general relevance. Therefore, we hope that some of these insights may be useful to researchers in the field who are interested in designing next-generation delivery agents. It should be noted that the mechanisms of cell penetration often raise ambiguities, which is a result of how complex membrane translocation processes are. We do not describe these ambiguities herein, as we focus instead on the results obtained in our hands. In this context, we apologize if the valuable work of others is not described. 

## 2. What Is dfTAT and How Does dfTAT-Mediated Delivery Work?

### 2.1. Structure and Activity of dfTAT

**Figure 1:**
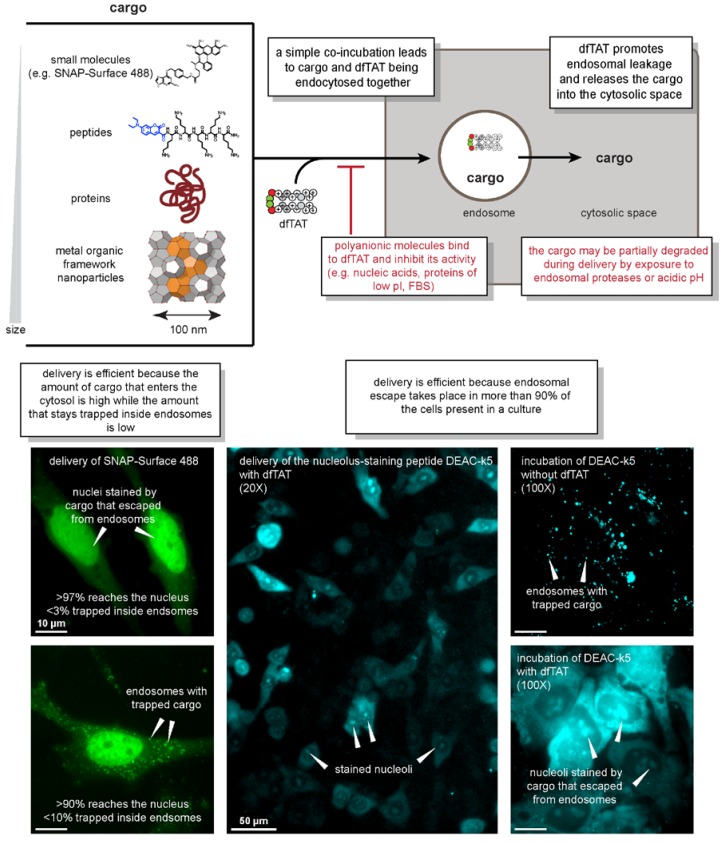
Macromolecular delivery by the endosomolytic cell-penetrating peptide (CPP) dfTAT in tissue cultures. dfTAT mediates cell penetration of various macromolecules by permeabilizing endosomal membranes efficiently. The stepwise process of cell entry—endocytic uptake followed by endosomal escape—is highlighted. Examples of cargos that have been successfully delivered are provided. Red boxes point to the limitations that are associated with this approach. Experimental evidence of the high efficiency of the delivery process is also shown in the form of microscopy images for two fluorescent cargos: the cell-impermeable small molecule SNAP-Surface 488 (which is delivered into cells expressing the nuclear tag SNAP-H2B), and the coumarin-labeled, nucleoli-staining peptide DEAC-k5. pI: isoelectric point; FBS: fetal bovine serum.

dfTAT is composed of two copies of the prototypical TAT peptide derived from the protein HIV-1 transactivator of transcription [22,23,24,25]. Both copies are conjugated to the fluorophore tetramethylrhodamine and are connected by a disulfide bond. When incubated with cells at concentrations of 5 µM or higher, dfTAT displays a cytosolic distribution, while its monomeric counterpart remains entrapped in endosomes. As shown in Figure 1, dfTAT can be co-incubated with a variety of cargos to promote their endocytic uptake. As dfTAT and cargo traffic into the endocytic pathway, the endosomolytic peptide mediates the permeabilization of endosomal membranes, thereby releasing the cargo into the cell. 

### 2.2. What Works

Using the simple co-incubation method, dfTAT has been successful at delivering cargos that are diverse in size, chemical properties, and function. These cargos include transcription factors, antibodies, and metal–organic framework (MOF) nanoparticles, as shown in Figure 1 [22,26]. Given their well-defined sizes (50–100 nm in diameter), successful MOF nanoparticle delivery indicates that dfTAT-mediated endosomal leakage involves membrane disruptions that are wide enough to accommodate large cargos. Moreover, the ability to introduce dfTAT and the cargo as separate entities enables the controlled titration of material into cells through the modulation of cargo concentration independent of dfTAT. This can be exceedingly difficult to achieve using other methods where the cargo must be covalently fused to the penetrating agent. One example of dfTAT’s versatility was the delivery of the transcription factor homeobox B4 (HOXB4) into cells. To evaluate the dfTAT-mediated delivery of HOXB4, a HOXB4-inducible promoter was used to regulate the expression of luciferase in cells. In the absence of dfTAT, luciferase expression remained completely suppressed. However, with dfTAT, varying levels of luciferase expression could be controlled based on the amount of HOXB4 titrated into the incubation mixture [22].

### 2.3. What Doesn’t Work

The polycationic nature of dfTAT is critical for its function (Figure 1). As described below, electrostatic interactions are important for cell penetration. Yet, these interactions can be abrogated if the charge of the peptide is screened in any way. For example, electrostatic interactions of dfTAT with negatively charged cargos (such as DNA) can cause aggregation, inhibiting the ability for dfTAT to induce endocytosis and endosomal leakage. This is also a consideration when delivery is performed in the presence of fetal bovine serum (FBS), which is a common supplement that is added to cell culture media. This is because FBS is rich in negatively charged albumin. Due to the binding of dfTAT to albumin, the introduction of FBS to the incubation medium reduces the activity of dfTAT in a manner that is proportional to the levels of FBS added [22].

### 2.4. Characteristics of the Delivery Process

The penetration of dfTAT, as observed by fluorescence microscopy, appears to be a binary event (Figure 1). In particular, after peptide incubation, two distinct cell populations can be observed: one with a diffuse fluorescence signal (with cytosolic and nucleolar staining, establishing that the signal is intracellular), and one with a punctate distribution, which is indicative of endosomal entrapment. The cells with a diffuse signal typically show very few puncta, and the cells with puncta show no diffuse fluorescence. At low concentrations of dfTAT, almost all of the cells show puncta only. As peptide concentration is increased, this population shifts toward cells of diffuse fluorescence, until cell penetration is achieved in close to 100% of the cells. Overall, this indicates that for any given cell, dfTAT penetration is either achieved efficiently, or not at all. This is in contrast to a situation where a uniform level of translocation would be achieved over the entire population. In this case, the concentration of peptide that is added controls how much gets in. Overall, these results instead indicate that the peptide activity is dependent on a concentration threshold [22,27]. This threshold may itself be dependent on multiple parameters (some of which are described below). Nonetheless, in practice, this means that small variations can have a big impact on delivery outcome, as they may shift the threshold of penetration. Conversely, using excess peptide concentrations well above the threshold required for successful penetration can alleviate this issue. 

The process of dfTAT-mediated delivery necessitates a minimum incubation period. dfTAT must be first taken up into the endocytic pathway. The peptide must then traffic to the organelles that constitute the specific site of membrane leakage (i.e., late endosomes, see Section 2). Using pulse-chase experiments, it was established that this process requires 10 to 45 min [22]. Based on these results, our typical protocol involves a 60-min incubation. Remarkably, this protocol does not lead to toxicity, it does not affect cell proliferation rates, and it does not impact the transcriptome [22]. This means that efficient delivery can be performed without inducing dramatic disturbances in the cell. 

For successful delivery to occur, dfTAT does not need to interact with its cargo. Instead, dfTAT and cargo simply need to traffic together within the endocytic pathway of a cell. In turn, this means that the delivery process must also be carried out by pre-incubation of the cargo first, and by adding dfTAT second. If the two incubation steps are performed immediately one after the other, dfTAT will “catch up” to the cargo within the endocytic pathway, and cell penetration of both peptide and cargo will be achieved. This can be particularly beneficial when attempting to deliver cargos prone to aggregation, as the peptide does not come into contact with the cargo until it reaches the same endocytic organelles as the pre-incubated cargo. In some cases, this two-step incubation process may therefore result in higher delivery efficiencies than a co-incubation protocol. However, it should be noted that if time is allowed to pass between the two incubation steps, dfTAT will penetrate cells, but delivery of cargo will fail. This is because the cargo may reach lysosomes before dfTAT may have a chance to cause endosomal leakage. 

In most of our experiments, dfTAT and cargo co-incubation leads to a pulse delivery of cargo into cells. This means that a high concentration of cargo may enter cells during a typical 60-min co-incubation. However, the cargo, which is now subjected to intracellular degradation, may soon vanish, depending on its intrinsic half-life. Yet, for many applications, a sustained cargo activity may be desirable (this is what is typically expected in DNA transfection experiment where gene products are continually produced by the cell). Notably, due to the low toxicity of dfTAT, it is also possible to repeat several dfTAT deliveries on the same cell population within a short time frame [22]. We have not established whether there is a limit to the number of delivery steps that can be performed on cells before toxicity arises. It is also unclear whether cells can sustain endosomal leakage for a prolonged period of time: cells may be able to sustain a burst of endosomal membrane leakage, but also may die if membranes are kept permanently permeable.

A limitation of using the endosomal pathway as a route of cell entry is related to degradation. Endosomes contain various hydrolases, and both dfTAT and cargo can be subjected to degradation while transiting within the lumen of these organelles. The degradation of dfTAT by endosomal proteases can significantly impact the membrane-leakage activity of the peptide, thereby diminishing delivery efficiencies [28]. Degradation of the cargo may vary as it is presumably cargo-dependent. In principle, unfolded peptides or proteins are more prone to degradation than their folded counterparts, and this should be a consideration when delivering such cargos into cells [29,30,31].

## 3. How Does dfTAT Get Inside Cells and Promote Endosomal Leakage?

### 3.1. Where Is Endosomal Escape Taking Place in the Cell?

**Figure 2:**
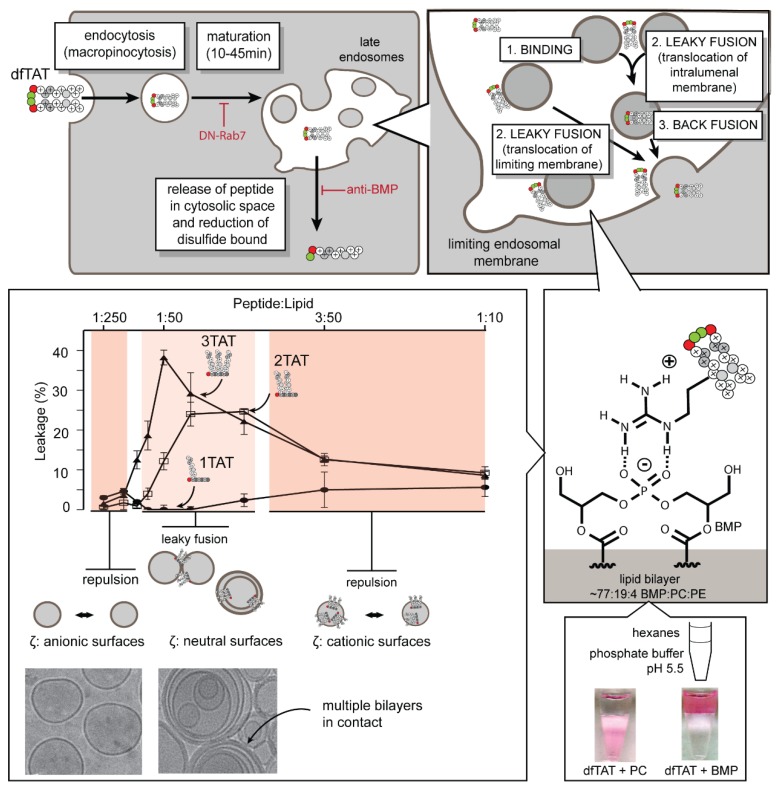
Model of how dfTAT causes the leakage of late endosomes through interactions with the anionic lipid bis(monoacylglycero)phosphate (BMP). dfTAT is taken up by the cell primarily via macropinocytosis. It is then trafficked along the endocytic pathway to late endosomes, which is a process that spans an approximate 10–45 min time window. dfTAT causes the leaky fusion of late endosomal membranes. The nature of this process remains unclear and may hypothetically involve the translocation at the limiting membrane of the late endosomes, or the translocation of intralumenal vesicles. In vitro, dfTAT selectively permeabilizes lipid bilayers that contain the late endosomal anionic lipid BMP. Permeabilization involves leaky fusion, which is a process where peptides displaying several CPP copies (e.g., 2TAT and 3TAT) can neutralize anionic bilayers and bring them into contact. Permeabilization may also relate to the unique ability of dfTAT and BMP to partition into hydrophobic environments. In particular, dfTAT partitions into hexanes with BMP, but not with the lipid phosphatidylglycerol (PG), which is a structural isomer of BMP (hexanes, as shown in the figure, mimic the hydrophobic tails of lipids). PC: phosphatidylcholine; PE: phosphotidylethanolamine.

Macropinocytosis is the dominant route by which dfTAT uptake occurs (Figure 2). Inhibitors of caveolae and clathrin-mediated endocytosis have little effect on dfTAT uptake. However, amiloride and bafilomycin, which are inhibitors of macropinocytosis and endosomal acidification respectively, reduce the delivery of dfTAT [22]. Thus, macropinocytosis and endosomal acidification are important for dfTAT penetration. Once in the endocytic pathway, dfTAT escapes from late endosomes to gain cytosolic access. The late endosome was identified as the gateway organelle in several in cellulo assays. For instance, the overexpression of the dominant-negative Rab-7 protein resulted in the inhibition of early to late endosome trafficking, and reduced dfTAT penetration. Additionally, preloading late endosomes with the peptide DEAC-k5 followed by the incubation of dfTAT results in the leakage of DEAC-k5 into the cytosol. However, dfTAT-mediated leakage of DEAC-k5 is not recapitulated when DEAC-k5 is sequestered in the lysosome, indicating that dfTAT likely escapes from late endosomes [27].

### 3.2. What Cellular Factors Are Involved in Endosomal Leakage?

dfTAT-mediated endosomal escape requires the late endosome-enriched lipid bis(monoacylglycero)phosphate (BMP) [27,32,33]. The incubation of cells with an anti-BMP mAb blocks dfTAT penetration, suggesting that BMP-containing membranes are involved in the endosomal escape of dfTAT (our data indicate that anti-BMP blocks the fusion of these membranes) [27]. The validity of this result was supported by several in vitro assays. In particular, dfTAT causes the leakage of liposomes that have a lipid composition mimicking that of late endosomes (77:19:4 BMP:PC:PE), but not of liposomes mimicking other cellular membranes [22,33]. This phenomenon requires membrane fusion (lipid mixing). It is observed with the lipid bilayer containing BMP, but not other anionic lipids, including phosphotidylglycerol (PG), which is a structural isomer of BMP. It is also observed with dfTAT, but not with its monomeric counterpart fTAT. As a matter of fact, as shown in Figure 2, leaky fusion increases with the number of CPP copies present in a construct (1, 2, and 3TAT constructs, as described in Section 3, are shown), and it coincides with the neutralization of the anionic bilayer by the cationic peptide (as determined by zeta potential measurements). Liposome-to-liposome contacts are then enabled by the peptides, and dramatic membrane restructuring can be observed (as shown in the cryo-microscopy, or cryo-EM, images that are presented in Figure 2). Yet, if an excess of peptide covers the surface of liposomes, liposomes display cationic surfaces, which lead to repulsions between liposomes and the subsequent inhibition leaky fusion. 

The uniqueness of how BMP interacts with dfTAT is further highlighted by a partitioning assay. When testing several different negatively charged phospholipid species, only BMP is capable of transferring dfTAT into an organic phase that mimics the hydrophobic environment of a lipid bilayer (Figure 2) [27]. This activity is presumably related to the overall inverted cone shape of BMP, which gives it fusogenic properties and allows for it to form inverted micelles [34]. The relevance of this activity in the endosomal leakage process is unclear. Yet, it is interesting to speculate that it may play an important role in the membrane restructuring ability displayed by dfTAT. Taken together, our current working model is that dfTAT, upon transport into late endosomes, first binds to BMP-containing intralumenal vesicles. This binding then results in a leaky fusion event that either allows for the direct translocation of dfTAT across the limiting membrane, or the release of dfTAT once the vesicle back fuses with the limiting membrane (Figure 2).

Although the dominant route by which dfTAT enters cells has been identified, the possibility that dfTAT enters through other pathways at less detectable levels cannot be ruled out. Since BMP is also present in lysosomes, it is possible that dfTAT leakage is occurring through this organelle as well [32,33]. However, our results indicate that such leakage is below the detection limit of fluorescence-based assays. Finally, it is possible that the peptide exploits other routes of cellular penetration, including direct plasma membrane translocation, if its concentration is increased. This is something that has been reported for other CPPs, but that we have not tested [35,36]. 

## 4. What Are the Features That Make dfTAT Active?

**Figure 3:**
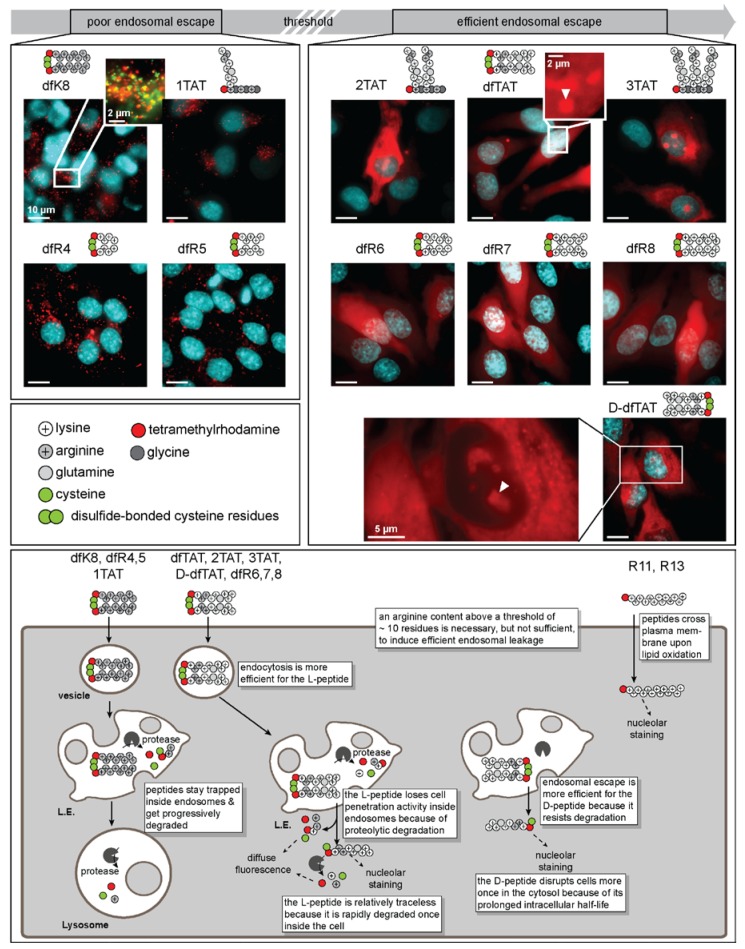
Active vs. inactive dfTAT variants. A threshold guanidinium density must be reached to achieve successful cytosolic penetration. Peptides such as dfK8, 1TAT, dfR4, and dfR5 do not meet the threshold guanidinium density, and as such, remained trapped within endosomes, as seen as puncta in the included fluorescence microscopy images. While trafficking through the endocytic pathway, these peptides become progressively more degraded. However, once a peptide has met or exceeded the guanidinium threshold, successful cytosolic penetration is obtainable (nucleolar staining is used to confirm that the fluorescence signal is intracellular and is highlighted with white arrows). This is shown as fluorescence microscopy images over 2TAT, dfTAT, 3TAT, dfR6, dfR7, and dfR8. Owing to susceptibility to proteolysis, l-peptides are degraded along the endocytic pathway. This results in a decreased activity upon reaching the late endosome. However, one favorable outcome of this characteristic is that upon successful cytosolic penetration, l-peptides are relatively traceless, as they are also rapidly degraded within the cell. Notably, peptides that are protease resistant (e.g., d-amino acid peptides) are less efficient at inducing endocytosis, but more efficient at causing endosomal escape relative to l-amino acid counterparts. These peptides remain intact and localize to the nucleoli of the cell for a prolonged period of time (>3 days; in contrast, l-peptides are fully degraded within 3 h, leading to a disappearance of nucleolar staining). As a drawback, d-peptides disrupt cells more once in the cytosolic space, owing to the prolonged intracellular half-life. Finally, linear, non-branched peptides that have a high guanidinium content (e.g., R11, R13) enter cells by direct plasma membrane translocation. In our hands, this process is dependent on culture conditions, membrane oxidation, and lipid peroxidation. Why branched and linear species differ in their internalization routes remains unclear. This nonetheless suggests that arginine content alone is not sufficient to confer endosomolytic activity, and that other chemical or structural features must be at play in compounds such as dfTAT.

dfTAT is composed of two copies of the tetramethylrhodamine-labeled peptide fTAT (sequence: CK(ε-NH-TMR)RKKRRQRRRG-NH_2_). The peptides are dimerized via a disulfide bond and, as such, can be disassembled into the monomeric units upon exposure to a reducible environment (e.g., the cytosolic space of a cell). dfTAT efficiently penetrates the cytosolic space of cells in a dose-dependent manner (Figure 3). However, this penetration activity is only observed when the peptide is dimerized. Whenever cells are treated with the non-dimerizable analog of fTAT, acfTAT, no cytosolic penetration activity is observed. Notably, the marginally higher endocytic uptake efficiency of dfTAT relative to acfTAT does not explain the disparity in cytosolic penetration. Taken together, these data suggest that the cytosolic penetration activity of dfTAT arises from its unique endosomolytic activity, as opposed to a difference in cellular uptake. Structure–activity relationship (SAR) studies have been conducted over analogs of dfTAT to investigate two unique molecular characteristics of the highly efficient CPP. These characteristics are the effect of guanidinium density (and, by proxy, charge density), as well as CPP multimerization on the cytosolic penetration activity.

### 4.1. Cytosolic Penetration Is Dependent on Reaching a Threshold Guanidinium Density

dfTAT has a total charge of +18 (stemming predominantly from 12 arginine residues and four lysine residues) and a charge density (charge/kDa) of +4.4. To address the effect that charge density has on cytosolic penetration, the peptides dfR8 and dfK8 were generated [37]. Each branch of these peptides contained an N-terminal TMR-labeled cysteine-lysine (CK) scaffold coupled to octa-arginine or octa-lysine, respectively. dfR8 and dfK8 have the same total charge as dfTAT, and by proxy, comparable charge densities. However, the cytosolic penetration activities of these constructs are different. dfR8 mirrored the cytosolic penetration activity of dfTAT, whereas dfK8 displayed much lower cytosolic penetration (<10%). These data suggest that charge density alone is insufficient to produce highly efficient cell-penetrating agents. Instead, successful cytosolic penetration relies on the density of guanidinium functionalization. When compared with dfTAT, it is important to consider that dfR8 contains four additional arginine residues. To determine the contribution of guanidinium density toward cytosolic penetration, the dfRn series of peptides were generated. Polyarginine chains of differing lengths (Rn where *n* = 4, 5, 6, or 7 Arg residues) were synthesized with a universal N-terminal CK(TMR) scaffold. The charge density of the dfR_n_ peptides remain relatively unchanged compared with dfTAT by truncating the length of the monomeric peptide (+3.7–4.4). To determine cytosolic penetration efficiency, the peptides were assayed at different concentrations [37]. The analogs with less guanidinium content (e.g., dfR4 and dfR5) yielded little to no cytosolic penetration activity at all of the tested concentrations tested. Yet, a drastic increase in cytosolic penetration activity (comparable to that of dfTAT) was observed for dfR6, dfR7, and dfR8. A threshold of >10 arginine residues is therefore required to achieve highly efficient cytosolic penetration. However, in constructs exceeding 12 total arginine residues (e.g., dfR7 and dfR8), increased cytotoxicity was observed. Taking into consideration both the cytosolic penetration and cytotoxicity data, 12 arginine residues (e.g., dfR6) represents a “sweet spot” for peptides to be highly efficient yet minimally cytotoxic.

### 4.2. Charge Density and Multimerization Play a Role in Successful Cytosolic Penetration

While the dfRn peptides were generated to isolate the effect of guanidinium content, charge density and multimerization remained constant. A complementary study was conducted utilizing the nTAT series of peptides in which the number of TAT copies was modulated to evaluate the contribution of multimerization and charge density on cytosolic penetration efficiency [38]. The nTAT peptides (1TAT, 2TAT, and 3TAT) were generated by synthesizing a TMR-KGKGKG scaffold with one, two, or three copies of the TAT peptide conjugated to the ε-NH_3_^+^ of each lysine of the scaffold. Each peptide only differed in the number of TAT copies, which led to differences in charge and guanidinium density. When used to treat cells, 1TAT was incapable of entering cells. In contrast, 2TAT penetrated the cytosolic space of cells, albeit with only a modest efficiency. Moreover, 3TAT exhibited efficient cytosolic penetration after treating cells with as little as 1 µM of peptide (Figure 3). Yet, while 1TAT and 2TAT were innocuous to cells, 3TAT was cytotoxic (>10% cytotoxicity) when cells were treated with ≥5 µM of peptide. When considering the cytosolic penetration and cytotoxicity data of the nTAT series in comparison with the dfRn series, there is a guanidinium threshold that is required to achieve efficient cytosolic penetration. As shown in Figure 2, this threshold enables the leaky fusion of BMP-containing lipid bilayers. Furthermore, these two datasets corroborate the idea that excessive arginine content leads to cytotoxicity.

### 4.3. Is There More to dfTAT Activity than Arginine Content?

The results of the in vitro assays show that a threshold guanidinium content is required for membrane leakage. Notably, 2TAT is far less active than dfTAT or dfR6, even though these three peptides meet the Arg threshold. The reason for this disparity in activity is not yet known. A difference between the peptides that could contribute to this disparity is the number of fluorophores that are incorporated into these constructs (one copy for 2TAT versus two copies for dfR6). In fact, a four to seven-fold decrease in cytosolic penetration and membrane lytic activity was observed for non-fluorescent variants of 2TAT and 3TAT. This suggests that the incorporation of fluorophore(s) enhances the cytosolic penetration activity of guanidinium-rich membrane lytic agents. Additionally, cell permeability is also affected by the how the guanidinium groups are displayed in a structure. For example, we have found that both R12 (a linear chain peptide of 12 arginine residues) and dfR6 are both cell-permeable. Yet, the linear representation (R12) leads to direct membrane translocation, whereas the dimerized dfR6 undergoes endocytic-mediated cellular internalization (Figure 3). In particular, we have observed that R12 translocates across the plasma membrane under conditions of membrane oxidation (e.g., when cells are grown at 20% oxygen, but not 2% oxygen, or when oxidants are present in growth media, but not when antioxidants are added) [36]. Therefore, linear peptides take a different route into cells than branched species.

### 4.4. Do Chirality and Protease Degradation Impact Cell Penetration?

In general, dfTAT penetration is a two-step process: endocytic uptake followed by endosomal escape. These parameters were investigated by utilizing an l- to d-amino acid conversion of dfTAT [28]. The d-amino acid variant of dfTAT (d-dfTAT) is resistant to proteolytic degradation (Figure 3). As a result, the levels of intact d-dfTAT that reach the late endosome are expected to be higher. It was found that the d-dfTAT analog achieved higher endosomal escape; however, it had reduced endocytic uptake. This is thought to be attributed to chirality-dependent interactions at the cell surface. In contrast, l-dfTAT follows an opposite trend with higher levels of uptake, but comparatively lower levels of endocytic escape due to degradation. This leads to a similar activity between the two analogs overall and highlights the importance of both steps in achieving successful cytosolic penetration.

It is expected that extensive membrane disruption and the simultaneous leakage of endosomal contents results in significant cytotoxicity [28]. Consequently, one might expect l-dfTAT to trigger a toxic response, given the mechanism of action. Surprisingly, the typical one-hour l-dfTAT incubation poses a paradigm shift in that it does not induce any observed toxicity or alterations in the cell proliferation rate [22]. Furthermore, whole genome microarray analysis revealed a remarkably small number of dysregulated transcripts. This is unprecedented, as previous examples of endosomal lysis impart cytotoxicity through the release of calcium and proteases into the cytosol [15,16]. This begs the question of how dfTAT is able to achieve such activity without lasting negative effects.

## 5. Conclusions

Efficient cell penetration by macromolecules requires the permeation of cellular membranes. In principle, it is often thought that the higher the efficiency of the cell penetration event, the more damaging the process is to membranes, and therefore, to the cell [39,40]. In other words, how does one let large molecules enter a cell without creating large holes in cellular barriers and without destroying these barriers? One of the surprising results obtained with dfTAT is that this general efficiency/toxicity paradigm is not necessarily true. The reason for this remains unclear. It is possible that the lack of a deleterious effect on cells treated with dfTAT is a phenomenon that is specific to late endosomal permeabilization. These organelles contain intralumenal vesicles, and it is possible that dfTAT and its cargos translocate across the membranes of these vesicles prior to entering cells via a back-fusion event. This in turn may sequester membrane damage within the interior of late endosomes, thereby avoiding the destruction of these organelles. Damage may also be masked by the engagement of membrane repair processes that rapidly attenuate the negative impact that membrane permeabilization may have on the cell. In addition, BMP has been shown to have unique fusogenic properties. This lipid may provide a unique fluidity to the late endosomal membrane, thus favoring transient and traceless translocation events. Finally, it should be noted that endosomal leakage can indeed be fatal to cells. As highlighted in our introduction, this is the case with CPP-mediated photochemical internalization (PCI), which is a process that leads to the rapid and toxic burst of calcium release from the lumen of endosomes to the cytosol and mitochondria of cells. In principle, calcium is also released from late endosomes, which are permeabilized by dfTAT. However, dfTAT-mediated cell penetration is a process that takes place on a scale of minutes, not seconds, as with PCI. Therefore, we envision that the leakage of calcium into cells during dfTAT treatment may be relatively slow and progressive, leaving cells time to re-establish homeostasis. Overall, regardless of why their permeation is tolerated, late endosomes appear to be a desirable and exploitable gateway into cells. 

Although the bottleneck of endosomal entrapment can be overcome, the cytosolic delivery of cargos mediated by dfTAT and dfTAT-like reagents has limitations and drawbacks. The electrostatic interactions between the polycationic CPPs and anionic BMP appear essential for endosomal escape. Yet, electrostatic interactions can take place with other anionic molecules, thereby contributing to the inhibition of cell penetration. For instance, dfTAT is ineffective at delivering polyanionic cargos, as delivery agent and cargo simply cluster and aggregate. Another consideration is peptide degradation throughout the endocytic pathway and within the cytosolic space of the cell. The degradation of peptides along the endocytic pathway leads to a decrease in endosomal escape activity, as degraded products lack the necessary arginine content to contribute toward endosomal escape. Conversely, peptides displaying protease resistance (e.g., d-dfTAT) remain active upon successful penetration into the cytosolic space. This poses an issue, as the membrane lytic CPP can potentially interact with anionic membranes from within the cytosolic space, yielding deleterious effects and even cytotoxic activity. In principle, such long-lasting CPPs may also interact with nucleic acids once inside the cells, contributing to defects in transcription and translation. Overall, this may therefore be a catch-22 of cell penetration: delivery reagents lose useful activity if they are not stable enough to resist the degradative environment of the lumen of endosomes but gain unwanted and undesirable activities if they persist in cells after delivery is achieved. 

Despite these limitations, arginine-rich peptides such as dfTAT provide immediate usefulness for many cell culture-based applications. Structure–activity relationship should continue revealing more insights on how dfTAT gains activity. Investigations into the mechanism of membrane disruption should also reveal the molecular underpinnings of membrane permeation. Together, this knowledge should facilitate the design of the next generation of CPPs. It may also provide insights on how to tune the activity of other delivery agents, including liposomes or polymeric nanoparticles, as these systems also often suffer from endosomal entrapment.

## References

[1] Brooks H., Lebleu B., Vivès E. (2005). Tat peptide-mediated cellular delivery: Back to basics. Adv. Drug Deliv. Rev..

[2] Heitz F., Morris M.C., Divita G. (2009). Twenty years of cell-penetrating peptides: From molecular mechanisms to therapeutics. Br. J. Pharmacol..

[3] Mager I., Eiriksdottir E., Langel K., El Andaloussi S., Langel U. (2010). Assessing the uptake kinetics and internalization mechanisms of cell-penetrating peptides using a quenched fluorescence assay. Biochim. Biophys. Acta.

[4] Madani F., Lindberg S., Langel U., Futaki S., Graslund A. (2011). Mechanisms of cellular uptake of cell-penetrating peptides. J. Biophys..

[5] Jones A.T. (2007). Macropinocytosis: Searching for an endocytic identity and role in the uptake of cell penetrating peptides. J. Cell. Mol. Med..

[6] Hayashi Y., Yamauchi J., Khalil I.A., Kajimoto K., Akita H., Harashima H. (2011). Cell penetrating peptide-mediated systemic sirna delivery to the liver. Int. J. Pharm..

[7] Lee Y.-J., Johnson G., Peltier G.C., Pellois J.-P. (2011). A HA2-Fusion tag limits the endosomal release of its protein cargo despite causing endosomal lysis. Biochim. Biophys. Acta.

[8] Johnson G.A., Muthukrishnan N., Pellois J.-P. (2013). Photoinactivation of Gram positive and Gram negative bacteria with the antimicrobial peptide (KLAKLAK)(2) conjugated to the hydrophilic photosensitizer eosin Y. Bioconjug. Chem..

[9] Wharton S.A., Martin S.R., Ruigrok R.W., Skehel J.J., Wiley D.C. (1988). Membrane fusion by peptide analogues of influenza virus haemagglutinin. J. Gen. Virol..

[10] Lee Y.-J., Johnson G., Pellois J.-P. (2010). Modeling of the endosomolytic activity of HA2-TAT peptides with red blood cells and ghosts. Biochemistry.

[11] Lee Y.-J., Erazo-Oliveras A., Pellois J.-P. (2010). Delivery of macromolecules into live cells by simple co-incubation with a peptide. ChemBioChem.

[12] Maiolo J.R., Ottinger E.A., Ferrer M. (2004). Specific redistribution of cell-penetrating peptides from endosomes to the cytoplasm and nucleus upon laser illumination. J. Am. Chem. Soc..

[13] Matsushita M., Noguchi H., Lu Y.F., Tomizawa K., Michiue H., Li S.T., Hirose K., Bonner-Weir S., Matsui H. (2004). Photo-acceleration of protein release from endosome in the protein transduction system. FEBS Lett..

[14] Selbo P.K., Weyergang A., Hogset A., Norum O.J., Berstad M.B., Vikdal M., Berg K. (2010). Photochemical internalization provides time- and space-controlled endolysosomal escape of therapeutic molecules. J. Control. Release.

[15] Srinivasan D., Muthukrishnan N., Johnson G., Erazo-Oliveras A., Lim J., Simanek E., Pellois J.-P. (2011). Conjugation to the cell-penetrating peptide tat potentiates the photodynamic effect of carboxytetramethylrhodamine. PLoS ONE.

[16] Muthukrishnan N., Johnson G., Lim J., Simanek E., Pellois J.-P. (2012). Tat-mediated photochemical internalization results in cell killing by causing the release of calcium into the cytosol of cells. Biochim. Biophys. Acta.

[17] Sung M., Poon G.M.K., Gariépy J. (2006). The importance of valency in enhancing the import and cell routing potential of protein transduction domain-containing molecules. Biochim. Biophys. Acta.

[18] Lönn P., Kacsinta A.D., Cui X.-S., Hamil A.S., Kaulich M., Gogoi K., Dowdy S.F. (2016). Enhancing endosomal escape for intracellular delivery of macromolecular biologic therapeutics. Sci. Rep..

[19] Arnusch C., Branderhorst H., de Kruijff B., Liskamp R.M.J., Breukink E., Pieters R. (2007). Enhanced membrane pore formation by multimeric/oligomeric antimicrobial peptides. Biochemistry.

[20] Van Baal I., Malda H., Synowsky S., van Dongen J.L.J., Hackeng T., Merkx M., Meijer E.W. (2005). Multivalent peptide and protein dendrimers using native chemical ligation. Angew. Chem. Int. Edit..

[21] Angeles-Boza A.M., Erazo-Oliveras A., Lee Y.-J., Pellois J.-P. (2010). Generation of endosomolytic reagents by branching of cell-penetrating peptides: Tools for the delivery of bioactive compounds to live cells in cis or trans. Bioconjug. Chem..

[22] Erazo-Oliveras A., Najjar K., Dayani L., Wang T.-Y., Johnson G.A., Pellois J.-P. (2014). Protein delivery into live cells by incubation with an endosomolytic agent. Nat. Methods.

[23] Frankel A.D., Pabo C.O. (1988). Cellular uptake of the tat protein from human immunodeficiency virus. Cell.

[24] Green M., Loewenstein P.M. (1988). Autonomous functional domains of chemically synthesized human immunodeficiency virus TAT trans-activator protein. Cell.

[25] Vivès E., Brodin P., Lebleu B. (1997). A truncated HIV-1 TAT protein basic domain rapidly translocates through the plasma membrane and accumulates in the cell nucleus. J. Biol. Chem..

[26] Lian X., Erazo-Oliveras A., Pellois J.P., Zhou H.C. (2017). High efficiency and long-term intracellular activity of an enzymatic nanofactory based on metal-organic frameworks. Nat. Commun..

[27] Erazo-Oliveras A., Najjar K., Truong D., Wang T.Y., Brock D.J., Prater A.R., Pellois J.P. (2016). The late endosome and its lipid BMP act as gateways for efficient cytosolic access of the delivery agent dftat and its macromolecular cargos. Cell Chem. Biol..

[28] Najjar K., Erazo-Oliveras A., Brock D.J., Wang T.Y., Pellois J.P. (2017). An l- to d-amino acid conversion in an endosomolytic analog of the cell-penetrating peptide TAT influences proteolytic stability, endocytic uptake, and endosomal escape. J. Biol. Chem..

[29] Authier F., Posner B.I., Bergeron J.J. (1996). Endosomal proteolysis of internalized proteins. FEBS Lett..

[30] Chatterjee B., Smed-Sorensen A., Cohn L., Chalouni C., Vandlen R., Lee B.C., Widger J., Keler T., Delamarre L., Mellman I. (2012). Internalization and endosomal degradation of receptor-bound antigens regulate the efficiency of cross presentation by human dendritic cells. Blood.

[31] Diment S., Stahl P. (1985). Macrophage endosomes contain proteases which degrade endocytosed protein ligands. J. Biol. Chem..

[32] Kobayashi T., Stang E., Fang K.S., de Moerloose P., Parton R.G., Gruenberg J. (1998). A lipid associated with the antiphospholipid syndrome regulates endosome structure and function. Nature.

[33] Kobayashi T., Beuchat M.H., Chevallier J., Makino A., Mayran N., Escola J.M., Lebrand C., Cosson P., Kobayashi T., Gruenberg J. (2002). Separation and characterization of late endosomal membrane domains. J. Biol. Chem..

[34] Falguieres T., Luyet P.P., Bissig C., Scott C.C., Velluz M.C., Gruenberg J. (2008). In vitro budding of intralumenal vesicles into late endosomes is regulated by Alix and Tsg101. Mol. Biol. Cell.

[35] Duchardt F., Fotin-Mleczek M., Schwarz H., Fischer R., Brock R. (2007). A comprehensive model for the cellular uptake of cationic cell-penetrating peptides. Traffic.

[36] Wang T.-Y., Sun Y., Muthukrishnan N., Erazo-Oliveras A., Najjar K., Pellois J.-P. (2016). Membrane oxidation enables the cytosolic entry of polyarginine cell-penetrating peptides. J. Biol. Chem..

[37] Najjar K., Erazo-Oliveras A., Mosior J., Whitlock M., Rostane I., Cinclair J., Pellois J.-P. (2017). Unlocking endosomal entrapment with supercharged arginine-rich peptides. Bioconjug. Chem..

[38] Brock D.J., Kustigian L., Jiang M., Graham K., Wang T.-Y., Erazo-Oliveras A., Najjar K., Zhang J., Rye H., Pellois J.-P. (2018). Efficient cell delivery mediated by lipid-specific endosomal escape of supercharged branched peptides. Traffic.

[39] Scharf B., Clement C.C., Wu X.X., Morozova K., Zanolini D., Follenzi A., Larocca J.N., Levon K., Sutterwala F.S., Rand J. (2012). Annexin A2 binds to endosomes following organelle destabilization by particulate wear debris. Nat. Commun..

[40] De Castro M.A.G., Bunt G., Wouters F.S. (2016). Cathepsin B launches an apoptotic exit effort upon cell death-associated disruption of lysosomes. Cell Death Discov..

